# Bioavailability of Orally Delivered Alpha-Tocopherol by Poly(Lactic-Co-Glycolic) Acid (PLGA) Nanoparticles and Chitosan Covered PLGA Nanoparticles in F344 Rats

**DOI:** 10.5772/63305

**Published:** 2016-01-01

**Authors:** Lacey C. Simon, Rhett W. Stout, Cristina Sabliov

**Affiliations:** 1 Department of Biological and Agricultural Engineering, Louisiana State University A&M and LSU Agricultural Center, USA; 2 Department of Pathobiological Sciences, Louisiana State University A&M, USA

**Keywords:** Alpha-tocopherol, Bioavailability, PLGA, Chitosan, Nanodelivery

## Abstract

It is hypothesized that the bioavailability of αT (alpha-tocopherol), an antioxidant, can be improved when delivered by poly(lactic-co-glycolic) acid (PLGA) nanoparticles (NPs) and chitosan covered PLGA nanoparticles (PLGA-Chi NPs), and that the mucoadhesive properties of chitosan may enhance absorption of αT. PLGA and PLGA-Chi NPs were characterized by measuring entrapment efficiency, size, polydispersity, and zeta potential. Nanoparticle physical stability, chemical stability of entrapped αT, and release kinetics were also measured. Pharmacokinetic studies were conducted by administering PLGA (αT) NPs, PLGA-Chi (αT) NPs, and free αT via oral gavage in rats. The size and zeta potential of the two particle systems were 97.87 ± 2.63 nm and −36.2 ± 1.31 mV for PLGA(αT) NPs, and 134 ± 2.05 nm and 38.0 ± 2.90 mV for PLGA-Chi (αT) nanoparticles in DI water. The particle systems showed to be stable during various in vitro assays. Bioavailability of nanodelivered αT was improved compared to the free αT, by 170% and 121% for PLGA and PLGA-Chi NPs, respectively. It was concluded that while chitosan did not further improved bioavailability of αT, PLGA NPs protected the entrapped drug from the GI environment degradation and proved to be an effective delivery system for αT.

## 1. Introduction

Alpha-tocopherol (αT), a free radical chain-breaking antioxidant, is the most bioactive form of vitamin E. When consumed by humans, it has been shown to provide numerous health benefits [29]. It faces the same challenges as other orally delivered lipophilic antioxidants including poor solubility in water, oxidative instability when exposed to heat, light, and oxygen, chemical and enzymatic instability in the gastro-intestinal (GI) tract, and low mucosal permeability [13; 22; 8]. In addition, the absorption, transport and distribution of αT is linked to dietary fat. Absorption in the intestine requires the presence of bile salts, pancreatic enzymes and adequate fat. Once vitamin E is internalized into the enterocyte, it is packaged into chylomicrons and enters the circulation via the lymphatic system. Once the chylomicrons are in circulation, their triglycerides are subjected to hydrolysis via lipoprotein lipase, which results in the transfer of lipids, including αT, to peripheral tissues [19]. Because of the connection between dietary fat and αT absorption, the bioavailability of αT is highly variable. Bioavailability, as defined by the FDA, is the rate and extent to which the active drug ingredient or therapeutic moiety is absorbed and becomes available at the site of action [13].

To address the variable, often low, bioavailability of αT, researchers have attempted different ways to improve its uptake by entrapping it into delivery systems. Nanoparticles (NPs) have a plethora of advantages over other delivery systems including the ability to protect drugs from GI degradation, prolong systemic circulation, reduce fed/fasted variable absorption, and control the drug's release [14]. It is therefore expected that with all the aforementioned advantages, bioavailability of orally delivered drugs in nanoform will be improved. PEGylated NPs have been developed for drug delivery [11; 3] and several studies have indicated that poly (lactic-co-glycolic) acid (PLGA) NPs have improved the bioavailability of drugs compared to their free form, such as cyclosporine (119% of free form) [16], estradiol (1014%) [20], doxorubicin (363%) [14], amphotericin B (793%) [14], curcumin (2583%) [14], (2200%) [33], (1560%) [17], and lutein [12].

Two mechanisms are proposed for improved bioavailability of nanodelivered drugs, expressed as the area under the curve (AUC) when plotting the concentration of the drug in the plasma as a function of time. Either the drug is transported within NPs as they are transcytosed, or the particles release the load in a controlled manner in the intestine, where it is efficiently absorbed. It is suggested that orally administered NPs travel through the GI tract where they are absorbed by endocytosis, or by lymphoid uptake, in the M-cells in the Peyer's patches [20]. Alternatively, the NPs may adhere to the M-cells and release only the entrapped drug into the cell. Mucoadhesive NPs are believed to improve the bioavailability of poorly absorbed drugs because of the strong attraction of the particles to the negatively charged mucosa of the intestine responsible for a longer residence time in the intestine [26]. Chitosan is a non-toxic biopolymer produced from the deacetylation of chitin, a component in crustaceans and fungi. It has recently become popular in the food and pharmaceutical industries for its beneficial characteristics including mucoadhesive properties, biocompatibility and biodegradability [4]. Chitosan can be used to confer a positive charge to PLGA NPs when deposited on the surface of the particles via electrostatic interaction, to form a mucoadhesive PLGA-chitosan (PLGA-Chi) NP [22; 2; 32]. One study showed a 228% increase in AUC when NPs made of PLA (polylactic acid) coated with chitosan were delivered to rats (Ishak et al., 2013).

The goal of this study was to determine: if PLGA and PLGA-Chi NPs improved the bioavailability of lipophilic antioxidants (αT) by reducing the variable absorption associated with differences in fat intake; and if the mucoadhesive properties of chitosan improved this bioavailability even more than PLGA NPs. It was hypothesized that PLGA and PLGA-Chi NPs improved the bioavailability of entrapped αT compared to free αT and, further, it was hypothesized that the mucoadhesive properties of PLGA-Chi increased the bioavailability of entrapped αT compared to αT delivered in PLGA NPs. To test the hypotheses, PLGA and PLGA-Chi NPs were orally administered to F344 rats and the pharmacokinetic profiles of the NP-delivered treatment compared to that of the control, or free αT. To explain the observed pharmacokinetic profile of the NP-delivered αT better, the release kinetics of αT from both NP systems were studied. In addition, the physical stability of the NPs and the chemical stability of the entrapped αT were examined under GI conditions.

## 2. Methods

### 2.1 Materials

Poly (D,l-lactide-co-glycolide) (50:50) with a molecular weight of 30,000-60,000 Da, Polyvinyl alcohol (PVA) (31,000-50000 Da), (±)-α-tocopherol (96%) and D-(+)-Trehalose dehydrate ≥99% were purchased from Sigma Aldrich (MO, USA). Chitosan with a molecular weight of 100-300 kDa was purchased from Fisher Scientific (NJ, USA) and ethyl acetate was obtained from Macron Chemicals (PA, USA). Nanopure water was obtained from Nanopure Diamond (IA, USA). Acetonitrile and methanol, HPLC grade, were obtained from EMD Chemicals (MA, USA). Acetic acid was purchased from Fisher Scientific (NJ, USA). The gastric media was made up of nanopure water, pepsin from porcine gastric mucosa (Sigma Aldrich) and hydrochloric acid purchased from Fisher Scientific with sodium chloride also purchased from Fisher Scientific (NJ, USA). The intestinal media was composed of nanopure water, pancreatin purchased from Sigma Aldrich and sodium hydroxide and potassium hydrogen phosphate both from Fisher Scientific. Pasteur pipettes and heparin tubes were both purchased from Fisher Scientific. Male F344 rats were purchased from Harlan (IN, USA). Bio-Serv (NJ, USA) provided both rat feed and corn oil stripped of tocopherols.

### 2.2 Nanoparticle synthesis

PLGA and PLGA-Chi particles with entrapped αT were synthesized by an emulsion evaporation method modified from Zigoneanu et al. [37]. First, the organic phase was made of 2.5 w/v% PLGA and αT at a 10 w/w% loading relative to PLGA mass dissolved in ethyl acetate. The aqueous phase was formed with 2% polyvinyl alcohol (PVA) dissolved in ethyl acetate-saturated nanopure water. The organic phase was then slowly added to 40 ml aqueous phase at an oil to water ratio of 1:5 v:v under continuous mixing. After the initial emulsification, the emulsion was subjected to three rounds of microfluidization by an M-110p lab homogenizer (Microfluidics, MA, USA) at 30,000 psi to reduce the size of the droplets. Once the emulsion was completely formed, the ethyl acetate was evaporated under vacuum in a rotovapor (Buchi R-124, Buchi Analytical Inc., DE, USA). To form PLGA-Chi particles, a 0.01 w/v% chitosan solution was made using nanopure water spiked with acetic acid, creating an acidic environment for proper chitosan solubility. The chitosan solution was added to a PLGA NP sample at a chitosan concentration of 10 w/w% relative to PLGA and mixed thoroughly using a magnetic spin bar. The final PLGA and PLGA-Chi NP suspensions were washed by dialysis with a 100 kD MWCO membrane suspended in 1.5 l water to remove the PVA, with water changes every eight hours for 72 hours. Finally, particles were lyophilized for 48 hours in the presence of trehalose as a cryoprotectant at a trehalose to particle ratio of 1:1.

### 2.3 Entrapment efficiency

The entrapment efficiency of αT in lyophilized NPs was determined by dissolving the powder (trehalose and particles) in 95:5 (v/v%) acetonitrile:water at a concentration of 0.2 mg mL^−1^. The solution was sonicated for one minute before incubation for two hours at ambient temperature. Following incubation, the sample was centrifuged at 30,000 rpm for 15 minutes in an Allegra 64R centrifuge (Beckman Coulter, Fullerton, CA, USA), then filtered with a 0.2 μm PVDF syringe filter. An Agilent 1200 HPLC (CA, USA) with a binary pump, autosampler and reverse-phase C18 Xorbax XDB column (Agilent, CA, USA) was employed for fluorescent detection of αT at an excitation and emission of 290 nm and 330 nm, respectively. The mobile phase involved 1 mL min^−1^ gradient flow of 10% water and 90% methanol, increasing to 100% methanol over eight minutes. The total analysis time was 21 minutes per injection. 25 μl of the filtered sample was injected by the autosampler and the area under the curve produced by the fluorescence detector was automatically integrated by Agilent Chemstation software Rev: B. 03.01 (317) (CA, USA).

A calibration curve with concentrations ranging from 6–0.1 μg/ml αT in 95:5 acetonitrile:water was produced to determine the sample concentration from the AUC. All linear curves used in this study had a correlation coefficient of at least > 0.99, and inter- and intra-assay variabilities were determined by quantifying n=3 replicates at five different concentrations of αT. The entrapment efficiency was determined by dividing the mass of αT per mg of powder by the theoretical mass of αT/mg powder.

### 2.4 Nanoparticle morphology, size, PDI, zeta potential

Particle morphology was identified by transmission electron microscopy (TEM) using a JEOL JEM-1400 (JEOL USA Inc., Peabody, MA) system. Lyophilized NPs were re-suspended in DI water, and one droplet of both PLGA and PLGA-Chi NPs in suspension was placed on a copper grid of 400 mesh with carbon film. Uranyl acetate (2%) was used as a stain, and the sample was dried before analysis. Size, polydispersity index (PDI) and zeta potential were measured by dynamic light scattering (DLS) using a Malvern Zetasizer Nano ZS (Malvern Instruments, Inc., MA, USA). For analysis, freeze-dried NPs were re-suspended in deionized water at a concentration of 0.1 mg ml^−1^. One mL of the particle suspension was measured in clear disposable zeta cells at 25 °C. The refraction index and viscosity were set equal to that specific to the DI water. The mean values of size, PDI and zeta potential were determined using a mono-modal distribution.

### 2.5 Nanoparticle physical stability

The pH stability of the PLGA and PLGA-Chi NPs was determined using the Malvern Zetasizer Nano ZS. Particles were suspended in nanopure water that was titrated from pH 2.5–9 in increments of 0.5 using dilute HCL and NaOH solutions [22]. The pH progression was designed to mimic the pH change during GI transit. Size and zeta potential were determined at each increment of pH 0.5 and one measurement per 0.5 pH increment was taken for both PLGA and PLGA-Chi NPs.

### 2.6 Alpha-tocopherol chemical stability

The chemical stability of αT was determined by exposing both PLGA and PLGA-Chi NPs with entrapped αT to physiological conditions and measuring the remaining αT present in the media. Specifically, the NPs were suspended at 3 mg NPs/ml in simulated gastric and intestinal media, and the suspensions were kept at 37 °C and 100 rpm. The simulated gastric media consisted of 3.2 mg/ml pepsin, 0.03M NaCl, and 7ml HCL/l media, all dissolved in nanopure water at a pH of 1.2. The intestinal media contained 10 μg/ml pancreatin, 0.05M kH_2_PO_4_ and 0.896 g NaOH/l media, all dissolved in nanopure water at a pH of 6.8. At various time intervals from 0–3 hours (gastric) and 0–72 hours (intestinal), a sample of the suspension was centrifuged for 90 minutes at 30,000 rpm, and the αT was extracted from both the pellet (NPs) and the supernatant (released αT). The amount of αT was determined using the HPLC method described in the Entrapment Efficiency section. The sum of the two quantities revealed the total amount of αT present; this number was compared to the total amount of αT determined by the entrapment efficiency at time 0.

### 2.7 Nanoparticle release kinetics

*In vitro* release profiles of αT from PLGA and PLGA-Chi NPs were determined under simulated gastric and intestinal environments outlined under the Chemical Stability section. Following preparation of the gastric and intestinal environments, PLGA and PLGA-Chi particles were suspended in the gastric or intestinal solution at a concentration of 3 mg/ml and placed in an incubator at 37 °C and 100 rpm. One ml samples were collected in triplicate at multiple time points over the course of the experiment, three hours under gastric and 72 hours under intestinal conditions. Samples were centrifuged at 30,000 rpm for 90 minutes to separate the particles. After particle centrifugation, αT was extracted from the collected particles as previously described for entrapment efficiency measurement.

### 2.8 Nanoparticle delivery media preparation

To confirm that PLGA and PLGA-Chi NPs effectively increased the bioavailability of entrapped αT compared to free αT, all treatments were delivered in the same media. Since the bioavailability of αT is dependent on the presence of dietary lipids, a special delivery system was made, keeping the amount of lipids constant. The delivery vehicle consisted of a flour-in-water slurry, made up of 300 mg/ml refined flour, which was combined with corn oil stripped of αT (Bio-Serv, NJ, USA) at a ratio of 30:70. The NPs or free αT were dissolved in this slurry before delivery. Each of the treatments included 1.5 mg αT (entrapped in NPs or in free form) delivered in the slurry in a one ml dose.

### 2.9 Experimental animals

All experimental protocols involving animals were approved by the Louisiana State University Institutional Animal Care and Use Committee (Baton Rouge, LA, USA) and were carried out in accordance with Directive 2010/63/EU. Male F344 rats (Harlan Laboratories, IN, USA) weighing 240 ± 6 g were housed two per cage and acclimated for one week with access to food and water *ad libitum*. During the last four days of the acclimation period and for the duration of the study, the rats were fed an AIN-93G rodent diet (Bio-Serv, NJ, USA), which was stripped of all tocopherols. The rats were fasted for 12 hours before treatment with free access to water, and n=6 rats were gavaged at time 0 for both treatments and control doses. After gavage, the tocopherol-free feed was replaced and the rats were allowed unlimited access. During blood collection, the rats were temporarily anesthetized with isoflurane. Blood was collected via the retro-orbital plexus over 72 hours after gavage. Three rats from each group were serially bled at 0, 2, 8, 24, 48 and 72 hours. The remaining three rats in each group were serially bled at 0, 4, 12, 36 and 72 hours. The collected blood was transferred to lithium heparin tubes (Fisher Scientific, NJ, USA) to prevent clotting, and the plasma was separated from the blood immediately after by centrifugation at 6,000 rpm for eight minutes at 20 °C. The collected plasma was stored at −80 °C until processing and analysis.

### 2.10. Alpha-tocopherol plasma concentration measurement

To determine the αT concentration, 25 μl of plasma was thawed and spiked with delta-tocopherol (dT) as an internal standard (IS). The final IS concentration was 0.1 μg/ml. Acetonitrile was added to the spiked plasma to precipitate the proteins at a 95:5 acetonitrile:plasma ratio. After, the mixture was sonicated for one minute in 1.5 ml eppendorf tubes and allowed to sit undisturbed for 2.5 hours. The samples were then centrifuged at 30,000 rpm for 15 minutes and the supernatant was filtered through a 0.2 μm PVDF syringe filter before being subjected to HPLC-MS analysis, as follows.

Electrospray analysis was completed using an Agilent 1200 liquid chromatograph with autosampler, in sequence with an Agilent 6210 mass spectrometer. The 6210 is an ESI – TOF (ElectroSpray Ionization – Time of Flight) instrument. Separation was accomplished using a Zorbax SB-C18 column (2.1 × 30mm, 3.5-Micron) (Agilent Technologies, Santa Clara, CA). The injection volume was 10 μl, with a mobile phase consisting of (A) water with 0.1% formic acid and (B) methanol with 0.1% formic acid at a flow rate of 0.2 ml/min. The gradient was 90% methanol and 10% water at time 0, then the methanol was increased to 100% after eight minutes until the run was over at 21 minutes after injection.

For purpose of data analysis, the following ions were extracted from the total chromatogram: 429-431 m/z for αT and 402-403 m/z for dT. Extracting these ions resulted in peaks that were integrated using the areas for analysis. A ratio was calculated by the area of the αT divided by the area of the dT using MassHunter version B.02.00.

The sample data were compared with a calibration curve prepared by spiking blank plasma samples with various amounts of a stock solution of αT (0.05, 0.1, 0.5, 1.0, and 2.0 μg αT/ml) and a constant concentration of 0.1 μg/ml (final) dT internal standard (IS). Both inter- and intra-assay variabilities were determined by quantifying n=3 replicates at five different concentrations of αT using the HPLC-MS method described for sample processing.

### 2.11. Pharmacokinetic analysis

The pharmacokinetic parameters were calculated using NCSS 9, version 9.0.7 (Kaysville, UT, USA). The trapezoidal rule was employed to determine the AUC for oral administration of both treatments and controls. The area under the concentration-time curve (AUC) was a means to quantify the total amount of drug (αT) that reached systemic circulation. The relative bioavailability used in this study (F) was defined as the AUC of the drug orally delivered by a specific system divided by the AUC of the drug orally delivered in free form. C_max_ represented the maximum concentration of the drug detected in the plasma, and T_max_ was the time at which this occured.

### 2.12. Statistical analysis

The statistical significance of the data was determined using SAS® version 9.3 (SAS Institute Inc., NC, USA). A mixed-model analysis of variance with a random mixed-effects procedure was used to estimate the significance of the all data and the difference between the means was presumed significant if the *p*-value was less than or equal to 0.05.

## 3. Results

### 3.1 Entrapment efficiency

The entrapment efficiency was determined by comparing the amount of αT present in the NPs after freeze-drying to the amount of αT used in the synthesis of the particles. The amount of αT measured in the PLGA NPs was 25.12 ± 2.6 μg/mg NPs, and the amount measured in the PLGA-Chi NPs totalled 19.49 ± 0.38 μg/mg NPs. According to the theoretical values, these measurements confirm a 95.4 ± 9.85% entrapment efficiency in the PLGA NPs and a 77.95 ± 1.51% entrapment efficiency in the PLGA-Chi NPs.

### 3.2 Nanoparticle morphology, size, polydispersity and zeta potential

**Figure 1.**
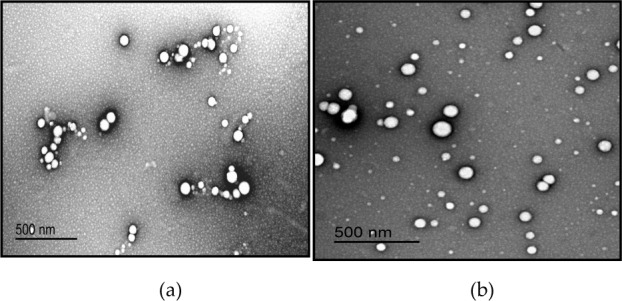
TEM images of PLGA NPs (a) and PLGA-Chi NPs (b)

The PLGA and PLGA-Chi NPs showed a spherical morphology when visualized by TEM (Figure 1). According to DLS analysis, the PLGA and PLGA-Chi NPs had an average diameter of 97.87 ± 2.63 nm and 134.1 ± 2.05 nm and a PDI of 0.156 and 0.298, respectively (Table 1). The zeta potential of freshly made PLGA NPs was −36.2 ±1.31 mV, while the zeta potential of the PLGA-Chi NPs was 38.0 ±2.90 mV when suspended in nanopure water at pH=5.5 (Table 1).

### 3.3 Nanoparticle physical stability

**Figure 2.**
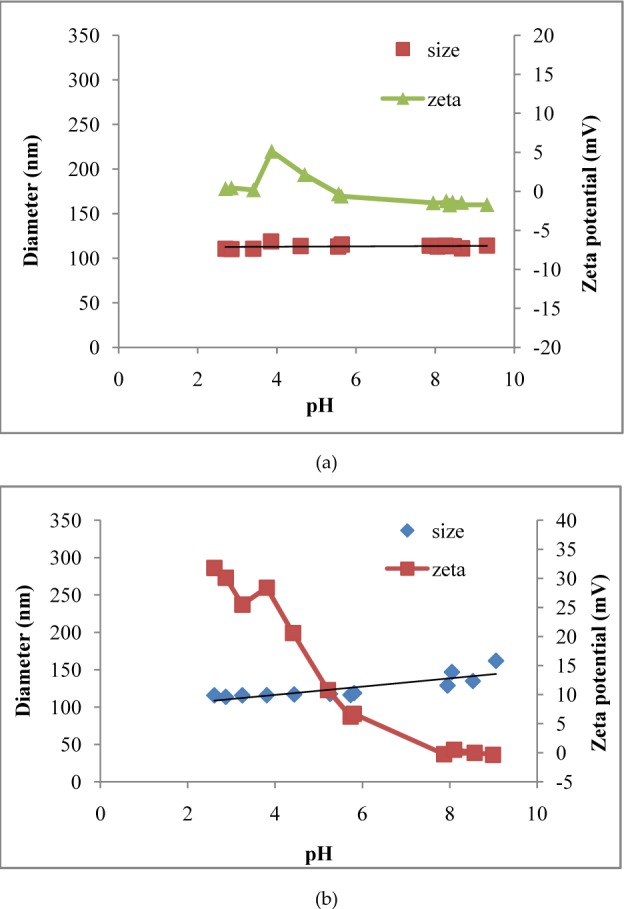
Change in size and zeta potential of PLGA (αT) NPs (a) and PLGA/Chi(αT) NPs (b) with respect to changing pH (pH 2.5–9)

The PLGA NPs maintained a constant size (∼110 nm) and a close to neutral zeta potential throughout the pH titration range (pH=2.5–9), (Figure 2a, Table 1). PLGA-Chi particles varied more in size and zeta potential than the PLGA NPs over the same pH range. As the pH increased the zeta potential of PLGA-Chi NPs fell, approaching neutrality, while particle size increased from 110 to 140 nm (Figure 2b, Table 1).

### 3.4 Alpha-tocopherol chemical stability

**Figure 3.**
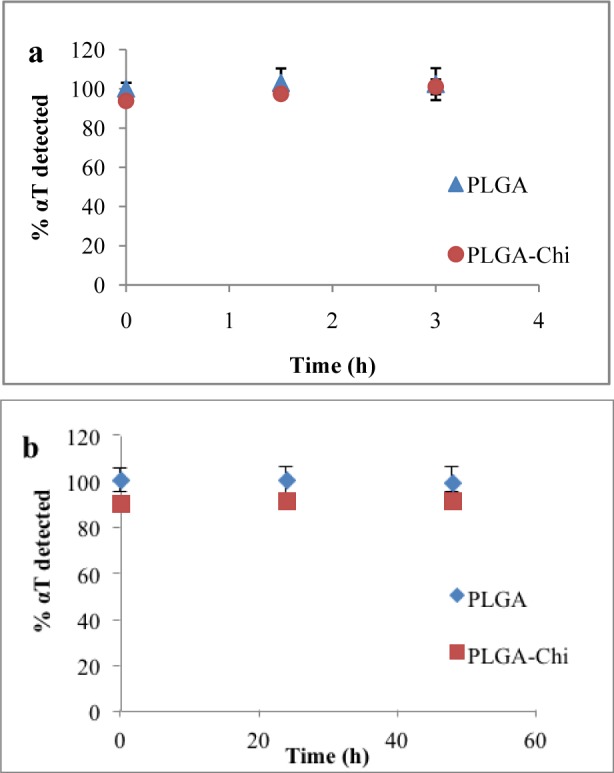
Total αT measured when PLGA and PLGA-Chi NPs were exposed to a simulated gastric environment for three hours (a) and simulated intestinal environment for 48 hours (b)

The results of the chemical stability experiment revealed no degradation of αT in the particles during the three hours they were exposed to the gastric environment. From time 0 to three hours, 100% of the entrapped αT was detected with both particle types (Figure 3a). Similarly, under simulated intestinal conditions, both particle types provided αT protection from degradation during the analysis time frame (48 hours) (Figure 3b).

**Table 1.** Summary of physical properties including size and zeta potential of PLGA and PLGA-Chi NPs under DI water and GI conditions

		DI water pH 5.5)	Gastric* environment (pH 2)	Intestinal* environment (pH 7)
PLGA NPs	size (nm) zeta (mV)	97.82 ± 2.63 -36.2 ± 1.31	110.3 0.35	114 -1.72
PLGA-Chi NPs	size (nm)	134.1 ± 2.05	116	138
	zeta (mV)	38 ± 2.90	31.8	−0.23

Note: * Data reported based on Figure 2

### 3.5 Alpha-tocopherol release kinetics

**Figure 4.**
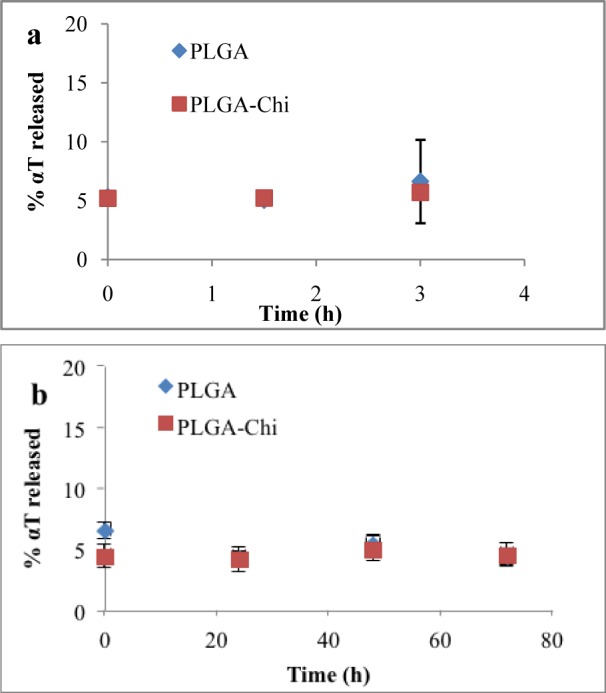
Percentage of αT released from PLGA and PLGA-Chi NPs in a simulated gastric environment at0, 1.5 and 3 hours (a) andin a simulated intestinal environment for 0, 24, 48 and 72 hours (b) as measured by HPLC After an acute administration of 1.5 mg αT delivered in PLGA NPs, PLGA-Chi NPs, and in free form, the concentration of αT in the plasma was measured over time (Figure 5). Free αT showed a rapid uptake and clearance after administration. The C_max_ was 2.91 μg/ml and occurred at a T_max_ of eight hours following oral gavage. At 12 hours, the plasma concentration of free αT approached the baseline. On the other hand, both NP-delivered systems showed a distinct uptake pattern compared to the free αT. At two hours after administration, an increase in plasma concentration was evident, but four hours later the concentration of αT delivered by both NP systems increased to over 3 μg/ml (Figure 6). These values stayed constant over the following eight hours, where the PLGA NP-delivered αT measured 3.35 μg/ml plasma and the PLGA-Chi NP-delivered αT measured 3.9 μg/ml plasma 12 hours after administration (Figure 6). Twenty-four hours after administration, the plasma concentration of αT delivered by both NP systems started to decrease. This continued until the baseline levels of αT were reached by αT delivered by both PLGA and PLGA-Chi NPs (Figure 5).

Approximately 5% of the entrapped αT was released at time 0. Three hours later, no more αT had been released from either PLGA or PLGA-Chi NPs in gastric media (Figure 4a). After 72 hours of exposure to the intestinal media, the 95% entrapped αT remained in the PLGA and PLGA-Chi particles. The release kinetics showed no differences in behaviour between PLGA and PLGA-Chi under these conditions (Figure 4b).

**Figure 5.**
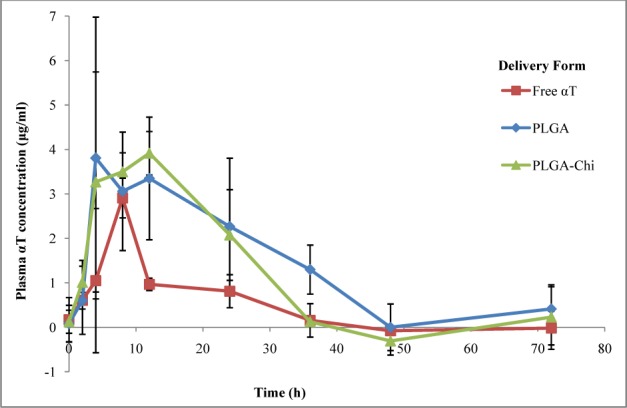
Plasma alpha-tocopherol concentration (μg/ml) delivered in PLGA, PLGA-Chi and in free form orally to F344 rats (n=6 per treatment). Three rats per treatment were bled at 0, 2, 8, 24, 48 and 72 hours while the remaining rats in each group were bled at 0, 4, 12, 36 and 72 hours. The concentrations were detected using positive ion mass spectroscopy.

**Table 2.** The pharmacokinetic parameters of T_max_, C_max_, and AUC were obtained for both treatments and the control from the plasma concentration-time curve (Figure 5) using NCSS 9 version 9.0.7 (NCSS, LLC. Kaysville, UT, USA)

Treatment	Tmax (h)	Cmax (μg/ml)	AUC (μg/h/ml)
Free αT	8	2.91	36.64
PLGA NPs	4-12*	3.81	99.00
PLGA-Chi NPs	12	3.92	80.93

From the plasma concentration-time curve, the AUC was obtained for each of the three curves (Table 2). These results indicated that the bioavailability of the treatment delivered by PLGA was 170% higher than the control. Similarly, the bioavailability of the bioactive delivered in PLGA-Chi NPs improved by 121% compared to the free αT. These values illustrated that both NP systems significantly improved the bioavailability of lipophilic bioactives when orally delivered in rats, but PLGA-Chi NPs did not improve the bioavailability compared with PLGA NPs under these conditions.

## 4. Discussion

Particle behaviour and the *in-vivo* fate of nanodelivered bioactives is not a result of one isolated parameter. Rather, it is heavily influenced by the interdependence of many parameters such as NP characteristics (e.g., size, surfactant, zeta potential), type of bioactive (e.g., hydrophobic or hydrophilic) and route of exposure (e.g., gastric, intestinal, blood).

Immediately after freeze-drying, the physical characteristics of PLGA and PLGA-Chi NPs showed similarities in size but differences in zeta potential. Specifically, they measured 97.87 ± 2.63 nm and 134.1 ± 2.05 nm for PLGA and PLGA Chi NPs, respectively. In terms of zeta potential, PLGA NPs were very negatively charged (−36 ± 1.31 mV) and PLGA-Chi NPs were very positively charged (38 ± 2.9 mV) when suspended in DI water at a pH of approximately 5.5. Although the differences in the particles were apparent in water, when they were exposed to GI conditions, the characteristics changed in a way that made both PLGA and PLGA-Chi NPs more similar than when in DI water. For example, when the particles were exposed to pH changes mimicking GI transit, the size in the gastric environment was 110 nm for PLGA NPs and 116 nm for PLGA-Chi NPs (Table 1). Under gastric conditions, the zeta potential of the PLGA NPs became close to neutral (0.345 mV), but the zeta potential of the PLGA-Chi NPs remained strongly positive (31.8 mV). On the other hand, the major characteristic separating the two NP systems when freshly made and suspended in water—the zeta potential—was no longer a factor under intestinal conditions. The zeta potentials of both particle systems reached neutrality, measuring −1.72 mV (PLGA NPs) and −0.229 mV (PLGA-Chi NPs) at an intestinal pH (Table 1). Therefore, under intestinal conditions NPs had a neutral zeta potential and a similar size, 116 nm (for PLGA NPs) versus 138 nm (for PLGA-Chi NPs) (Table 1).

Furthermore, knowledge of stability of the αT entrapped in the particles when exposed to simulated GI environments is required to understand the fate of the nanodelivered αT *in vivo*, especially when determining its bioavailability. No difference in protection of αT was demonstrated between PLGA or PLGA-Chi NPs when particles were exposed to simulated gastric and intestinal conditions over 48 hours. Over the time frame of this experiment, it was apparent that the release was in the early stages of the diffusion phase and no degradation of the polymer occurred, as supported by other studies [9; 24]. Alpha-tocopherol is a non-polar compound, so its tendency to diffuse from inside the lipophilic matrix of PLGA into a polar environment is not likely, explaining the low rate of release observed. It was apparent that αT was protected by the NP systems in the simulated GI environments (Figure 3) and no αT was released from the particles between time 0 and three hours (gastric) and 0 and 72 hours (intestinal) (Figure 4). From these findings, it must be concluded that the αT was transported to the intestinal barrier in an entrapped form and any improvement in bioavailability should be correlated to the enhanced transport of αT by the particles rather than the time release of the bioactive.

In general, the AUC of αT increased for other delivery systems reported in the literature. For example, when the AUC of the αT delivered to rats in free and nano-emulsion systems were compared, the nano-emulsion delivered αT showed a 143% increase in bioavailability compared to the control (free) form [15]. The AUC of αT was 18% higher compared to αT delivered in free form when entrapped in a calcium-pectinate microparticle system and delivered to rats [29]. A two-fold increase in αT bioavailability was achieved in humans when delivered as Gelucire 44/14 product [5].

The PK profile in Figure 5 confirmed that nano-delivery improved bioavailability of αT by more than 100% (*p* <0.05) when delivered with PLGA NPs. Perhaps the pharmacokinetic profiles of the free αT versus the NP entrapped αT provide insight as to why the AUC for the NP entrapped αT was larger. Specifically, the plasma concentration of free αT reached its maximum eight hours after administration (Figure 5). At 12 hours after administration, a rapid decrease in plasma concentration was observed, unlike the αT delivered in nano form. The PK curve for the αT delivered in nano form reached its maximum plasma concentration between four and 12 hours after administration. This plasma concentration gradually decreased, reaching baseline 48 hours after administration. Many factors can influence absorption and transport of lipophilic bioactives. Nano-delivery enhances the ability of a compound to be more easily transported transcellularly and paracellularly across the epithelial cells, whereas larger aggregates or microemulsions of the same bioactive might not be small enough to achieve the same level of transport. This phenomenon might be the reason we saw a much larger (over 100%) increase in plasma concentration of αT when delivered in nano form verses free form (Figure 5). In contrast to the hypothesis that the mucoadhesive properties of chitosan will enhance the uptake of αT, we saw no statistical difference in AUC between PLGA (αT) and PLGA-Chi (αT). When looking at the *in vitro* characteristics of the two particle systems exposed to simulated GI environments, it is evident they behave similarly (Table 1). We might also consider the possibility that any mucoadhesive properties displayed *in vivo* by the PLGA-Chi NPs might have been counteracted by the increase in size compared to PLGA NPs (Table 1).

No statistical difference was found between the PLGA and PLGA-Chi delivered αT (*p* > 0.05), in line with results published by Navarro et al, showing a similar NP biodistribution for PLGA and PLGA-Chi NPs in a F344 rat model [23]. Improved bioavailability of the nanodelivered drug was seen in other studies using PLGA NPs as a delivery system for different drugs [20; 17; 16] as well as for αT with other types of delivery systems [29; 5; 15].

## 5. Conclusions

The lack of data on orally delivered PLGA NPs presented an opportunity to investigate the ability of PLGA NPs to improve bioavailability of a model hydrophobic bioactive (αT) when delivered orally in rats. In addition, it was an opportunity to improve PLGA NPs' ability to transport drugs by adding chitosan, a mucoadhesive compound, on the surface of the particles. The results indicated that both particle systems were able to improve the bioavailability of the αT by over 100%. However, PLGA-Chi NPs did not improve the bioavailability of the αT delivered over that delivered by PLGA NPs. This was likely due to the similar properties of PLGA and PLGA-Chi NPs under the simulated intestinal environment, where absorption occurs. However, through the PK study, the effectiveness of PLGA NPs to improve bioavailability of an entrapped bioactive was demonstrated. PLGA NPs were proven eligible as a delivery system that can protect the bioactive from degradation by preventing release and maintaining its size stability in GI conditions. PLGA and PLGA-Chi NPs were not only efficient drug carriers, but they offered an abundance of advantages over other delivery systems in addition to improving bioavailability. Unlike other delivery systems, polymeric NPs such as PLGA NPs are capable of protecting the entrapped drug from the GI environment, can be tailored to desired release properties and are geared towards the size-dependent uptake suited to the intestinal membrane. PLGA NPs are a versatile, practical, and effective delivery system for hydrophobic bioactives with poor bioavailability.

## 6. Compliance with Ethical Research Standards

The authors declare no conflicts of interest. All research on animal subjects presented in this research was conducted in accordance with national and institutional ethical research standards for the care and use of laboratory animals.
